# Evaluation and Behavior of Spanish Bodybuilders: Doping and Sports Supplements

**DOI:** 10.3390/biom9040122

**Published:** 2019-03-28

**Authors:** Antonio Jesús Sánchez-Oliver, Moisés Grimaldi-Puyana, Raúl Domínguez

**Affiliations:** 1 Human Motricity and Sports Performance Area, University of Seville, 41004 Seville, Spain; sanchezoliver@us.es; 2 Department of Physical Education and Sports, Faculty of Educational Sciences, University of Seville, 41013 Seville, Spain; mgrimaldi@us.es; 3 Faculty of Health Sciences of Universidad Isabel I, Universidad Isabel I, 09004 Burgos, Spain; raul.dominguez@ui1.es

**Keywords:** sports supplements, harmful substances, doping, bodybuilding

## Abstract

The use of doping agents has these days become a public health problem, as it also affects young and non-competitive amateurs in different sports. To prepare for competition, bodybuilders perform aggressive dietary protocols, so, bodybuilders frequently consume nutritional supplements (NS) and banned substances in large dosages. Thus, the aim of this study is to analyze the prevalence of banned substances consumption and NS intake in competitive level bodybuilders. A total of 48 bodybuilders (44 males and 4 females) completed a validated online questionnaire on NS consumption. The quantitative data was presented as a mean (M) ± standard deviation (SD), as well as having minimum and maximum values. The categorical variables were expressed using frequencies and percentages. 83.3% of the participants declared that they had consumed or would consume banned substances, the most consumed being anabolic steroids (72.9%). One hundred percent of those sampled use NS. Whey protein (96%), branched-chain amino acids (BCAA) (94%), creatine (85%) and vitamin complexes (83%) were the most consumed, however, there is a low consumption of certain NS which could also increase athletic performance.

## 1. Introduction

Physical activity, exercise and sports are broadly encouraged by governments and health authorities due to their unquestionable benefits for health and the quality of life [1]. In this way, the World Health Organization (WHO) considers that the maximal oxygen uptake (VO_2max_) is the most important parameter in the functional capacity [2]. Low values of VO_2max_ increase the risk of suffering hypertension, diabetes and metabolic syndrome [3]. Besides endurance training, resistance training has demonstrated beneficial effects for increasing athletic performances and improving quality of life [4]. It is considered beneficial for various pathologies such as dyslipidemia, diabetes, hypertension, cancer, fibromyalgia, multiple sclerosis and Parkinson’s disease [5]. Therefore, endurance and resistance training are included in exercise programs for improving health and fitness [6].

Bodybuilding is one of the sports that prioritizes resistance training in its training program. A goal of performance for bodybuilders is to develop muscular hypertrophy and to obtain low fat levels based on a balanced body shape [7]. To obtain their performance objective, bodybuilders consume a hypercaloric diet during off-seasons to increase their muscle mass to the maximum possible and, in the competitive season, reduce their body fat the maximum amount possible to obtain the highest definition before competition [8]. To prepare for competition, bodybuilders perform aggressive dietary protocols [9] which may lead to a loss of bone mineral density, depression, a deficit of micronutrients, an obsession for food or even a decrease of the libido [10,11]. Furthermore, given that energy restriction prior to a competition has a negative effect on anabolic hormones, it can result in a reduction of the serum concentrations of hormones such as testosterone, insulin-like growth factor-1 (IGF-1) and insulin [12]. Moreover, this situation can be aggravated, as bodybuilders declare they also intake numerous nutritional supplements (NS) which have a different scientific basis [13]. In addition to the use of NS, this can result in a risk of inadvertent doping [14]. Bodybuilders declare that they consume banned substances [15,16] which fall into the category of “doping” because they are included in the list of prohibited methods and substances published by the World Anti-Doping Agency (WADA) [17]. The aim of the consumption of banned substances is that many of them increase adaptations to physical training and physical performance. So, bodybuilders sometimes abuse banned substances such as diuretics, stimulants, or anabolic substances [15,18,19,20,21]. Previous reports that have analyzed the prevalence of use of anabolic steroids suggest polypharmacy and high doses of injectable agents [16].

Furthermore, we must take into account that some banned substances are prohibited according to the WADA code depending on the sport, since they can produce advantages or are dangerous for the consumer in some sports and not in others. Every year WADA issues a list of countries and sports organizations which comply with WADA’s anti-doping code and are subject to its tests. Yet, it must be kept in mind that many professional sports, such as American football, and some federations, such as that of boxing, as well as many sports or sports physical activities such as bodybuilding, which is our case, do not comply with WADA’s code and its anti-doping regulations [22].There are limitations to obtaining precise data about the prevalence of consumption of banned substances. Current scientific evidence suggests that the consumption of NS is within a wide range that goes from 30 to 95% and which varies according to the context [23]. The use of banned substances by athletes and the non-athletic population is estimated at 1–5% of the population and over 50% in some sports modalities, with a greater use in men than women [24,25]. The use of doping agents, once restricted to professional athletes, has these days become a public health problem, as it also affects young and non-competitive amateurs in different sports [26,27] and entails considerable risks for health that include, among others: cardiovascular illnesses, diabetes, cancer, mental health problems, virilization in women and the suppression of the androgens produced naturally in men [24]. 

Given that bodybuilders frequently consume banned substances in large dosages [15,16,18,19,20,21] and the lack of research which has analyzed the prevalence of the consumption of banned substances in competitive level bodybuilders, the aim of this study is to analyze the prevalence of banned substances consumption and NS intake in competitive level bodybuilders.

## 2. Materials and Methods

The participants of this investigation were national and international level competition bodybuilders. The requirements for participating in this study included having been federated in bodybuilding at a national level for a minimum of 0.5 years and having participated in a national or international competition in the 0.5 years before the start of the study. For the recruitment of the sample, all the Spanish bodybuilding and fitness associations sent an informative email to athletes who fulfilled the inclusion criteria, informing about the characteristics of this research and inviting them to collaborate. We sent a validated online questionnaire to all the subjects who responded positively to the invitation to participate in the study. The questionnaires were answered anonymously and with prior consent by each participant, this study being approved by a local Ethics Committee.

All the athletes who voluntarily participated in this research filled out a validated online questionnaire on NS consumption. Its content was validated, guaranteeing the characteristics of the format and the instrument´s appearance; the capacity of its structure, the questionnaire’s questions, sequence and the response scale proposed; and its applications, prior an analysis of its shortcomings and benefits after revising the instruction of the instrument [23]. The instrument is divided into three sections: (Section 1) includes information about personal, social and anthropometric data; (Section 2) the aim of this section is to analyze information about sports activities and their contextualization; (Section 3) collecting information related to NS (including banned substances) consumption and the impact of this consumption on health. Finally, the survey included questions about the intake and the participants’ perceptions of banned substances. This survey was approved and has scored a methodological quality of 54% in Knapik et al.’s review [28].

The quantitative data was presented as a mean (M) ± standard deviation (SD), as well as having minimum and maximum values. The categorical variables were expressed using frequencies and percentages. With regards to NS, the frequencies of consumption was carried out based on the Australian Institute of Sport’s (AIS) ABCD system [29], a classification which divides NS into different groups according to the level of scientific evidence, where group A corresponds to NS with strong scientific evidence for use in specific situations in sport (including sports food, medical supplements and performance supplements), group B corresponds to emerging scientific support which merits further research (food polyphenols, amino acids, antioxidants, and others), group C corresponds to scientific evidence not supporting advantages amongst athletes and group D corresponds to substances banned or at a high risk of contamination that could lead to a positive doping test. The data were analyzed by the statistical analysis program SPSS version 22.0 (SPSS Inc., Chicago, IL, USA).

## 3. Results

A total of 61 bodybuilders completed the questionnaires, but finally, only 48 subjects (44 males and 4 females) were included in the study because 13 questionnaires were not appropriately filled out. At the sociodemographic level, the majority of the sample is non-students (64.6%), having higher education professional studies (29.2%), who are employees (41.7%), and who regularly carry out physical activity (100%). Training and bodybuilding competition characteristics are presented in Table 1.

Out of the respondents, 95.8% stated that they are following a diet. The diets most used are those considered high protein diet (16.7%) and Mediterranean (16.7%). The main reasons why they follow a diet are: aesthetics (52.1%) and health (25%). Concerning the opinion of the participants about NS consumption, 100% of them are in favor of NS use and all the sample declared that they consume NS. The mean NS consumption is 19.27 (±9.99) supplements. Seeking a greater sports performance (81.25%) and an improvement of physique (43.75%) are the two main reasons for consuming NS. The NS most consumed by the respondents are shown in Table 2. Those most used were: whey protein (96%), branched-chain amino acids (BCAA) (94%), creatine (85%) and vitamin complexes (83%).

83.3% of the participants declared that they had consumed or would consume banned substances. The data of banned substances consumed are shown in Table 3. Analyzing the goal of the consumption, the main reason is to improve their sports performance (87.51%). Finally, 68.8% of the respondents consider that the use of banned substances in the bodybuilding world is 5 (score out of 1 to 5), and the remaining (31.2%) 4 (score out of 1 to 5).

## 4. Discussion

The main result of this research is the high prevalence (83.3%) of banned substances in competitive level bodybuilders, in line with previous research [20,21]. As in other studies [16], we have detected an abuse of anabolic steroids as the most consumed banned substances (72.9%). Although the cases that come to light create awareness through the mass media, the quantification of the real use of banned substances by athletes is very complex. Nevertheless, in 2013 WADA reported that 2.1% (n = 5962) of the total samples analyzed (n = 269,878) resulted in an "adverse analytical finding" or an "atypical finding". Of these, 63.0% were anabolic substances (data which coincide with this study); 10.1% were stimulant agents; 7.5% were masking agents, such as diuretics; 6.3% were glucocorticosteroids; 3.8% were growth factors, peptide hormones and similar substances; and 3.6% were cannabinoids; the rest of the categories caused <3% of the atypical or adverse findings, the total of all these being approximately 6% of all the results obtained [24]. Comparing our results with the data of WADA, surveys can be useful to analyze the prevalence of banned substances consumption, at least in sports modalities such as bodybuilding where athletes are not submitted to antidoping tests, but their main limitations are related with the differences in the definitions of doping and banned substances for social reasons other than performance improvement, as well as trust in the honest self-reporting of an illicit activity [24].

Analyzing the goal of banned substances, our results are similar to NS intake. Seeking a greater sports performance, along with a better appearance, are the main reasons why banned substances are consumed, as occurs in the bibliography reviewed [16,26,30,31]. An alternative to banned substances consumption is that of NS, given that some NS have demonstrated strong scientific evidence about their ergogenic effect on sports performance [32]. In this sense, our results reflect that competitive level bodybuilders have a high consumption of NS that could have an ergogenic effect on their performance. For example, 96% of participants consume whey protein, 85% creatine, 83% complex vitamin and 75% caffeine.

The intake of post-exercise protein stimulates the synthesis of myofibrillar protein and a positive nitrogen balance [33], but only the essential amino acids have demonstrated an improvement of myoprotein synthesis [34]. Thus, whey protein is more effective that another type of protein, such as soya, for this positive effect [35]. Many bodybuilders have diets with a low quantity of micronutrients in precompetitive and competitive diets and complex vitamins and minerals could be beneficial to avoid micronutrient deficits [13]. In addition, the consumption of creatine (a prevalence of 85%) and caffeine (a prevalence of 75%) could be considered as ergogenic aids in bodybuilding. Creatine supplementation increases the restauration of phosphocreatine [36], the main energetic substrate in maximal efforts with a duration of less than 30 s [37]. Given that phosphocreatine depletion could be a limiting factor in bodybuilding and with the evidence of the ergogenic effect of this supplement to improve hypertrophy and strength [38,39], bodybuilders can benefit from creatine supplementation. Caffeine, for its part, is a potent stimulator of the sympathetic nervous system, improving the recruitment of motor units, the Na^+^–K^+^ pump response and the rate of calcium release from the sarcoplasmic reticulum [40]. These effects explain the ergogenic effect of caffeine supplementation on strength and power performance in resistance training [41].

In addition to previous NS, bodybuilders could improve their sports performance with other NS with a lower prevalence of consumption, such as B-alanine (52.1%), sodium bicarbonate (12.5%) or beetroot juice (6.25%). Dietary intake of B-alanine is the limiting factor of muscle carnosine synthesis [42], so prolonged B-alanine supplementation increases muscle carnosine levels [43]. The increase of carnosine levels can improve performance in efforts with a high glycolytic contribution (like bodybuilding) by improving the calcium bioavailability at the sarcoplasmatic level, by transporting calcium from the sarcoplasmic reticulum at sarcoplasm, while regulating intramuscular pH, by transporting an H^+^ to the cell membrane [44]. Thus, it has been demonstrated that B-alanine supplementation, in combination with creatine, increases the training load and levels of lean mass with respect to the exclusive intake of creatine [45]. Furthermore, it has recently been reported that supplementation with BA increases both 1 RM and the maximum power levels at 1 RM loads and maximum power levels after a period of supplementation in combination with resistance training [46]. In the same way, sodium bicarbonate supplementation improves extracellular pH regulation. Sodium bicarbonate supplementation has been shown to have a greater training volume in a resistance training session with hypertrophy orientation [47], as well as in a series with the maximum number of possible repetitions with a load corresponding to 80% of 1 RM [48]. Beetroot juice supplementation, due to its high content of nitrates (NO_3_^−^), is effective for increasing nitric oxide (NO) levels [49]. NO has important metabolic and hemodynamic functions, such as vasodilatation, an increase of muscular perfusion, gaseous exchange mitochondrial efficiency and enhanced muscle contraction [50]. In this way, beetroot juice supplementation has demonstrated improving power during dynamic contractions [51,52] and has been proposed as an ergogenic NS in resistance training [53].

The adverse effects of the use of NS may be due to factors such as safety and/or composition, as well as inappropriate use by athletes [32]. Among the different possible negative effects associated with the use of NS are: positive, voluntary or involuntary, in a doping test, sports performance impairment and/or adverse health effects [54]. Thus, it is important to know that NS can contain harmful and doping substances among which stimulants, estrogenic compounds, diuretics and anabolic agents are found, including anabolic and androgenic steroids, design steroids and prohormones [55], and even the presence of heavy metals such as mercury in whey protein supplements [56].

The data gathered in a study carried out by Geller et al. (2015), in which it is indicated that each year there are more than 23,000 emergency visits due to adverse events related with NS of which 9% end in hospitalization [57]. A more appropriate approach is to ask if the NS or ergogenic help considered really fulfills the three indispensable requirements of effectiveness, safety and legality [58,59,60]. Taking this into account, to foster the effective use of NS, maximize the taking of nutrients coming from foods to minimize or suppress NS use and, perhaps most importantly, to control and report on the great risks of the consumption of banned substances are the areas of education most needed in this environment.

An adequate and responsible use of supplementation by athletes and the professionals who advise them is essential. The knowledge about NS is fundamental due to the importance acquired by NS in the performance and health of the athletes. This knowledge must go beyond the effectiveness of NS, focalizing in the safety and legality of NS, since these three aspects (efficiency, safety and legality) produce most of the existing problems in relation to supplements on the sport [61].

## 5. Conclusions

Bodybuilders of competitive level present a high consumption of banned substances (83.3%), including, as in previous studies, a higher consume of anabolic steroids. Analyzing the NS consumption, we have found an elevated consumption of NS, such as whey protein, creatine, complex vitamin and caffeine, which could increase sports performance. However, we have registered a low intake of NS, like B-alanine, sodium bicarbonate or beetroot juice, which could also increase their sports performance.

## Figures and Tables

**Table 1:** Training and bodybuilding competition characteristics of the participants.

Measure	Average (± SD)	Range (Minimum–Maximum)
**Age (years)**	28.88 (8.1)	16.00–46.00
**Years associated**	2.96 (3.4)	0.50–15.00
**Weekly training days**	5.48 (0.9)	3.00–7.00
**Daily training hours**	1.73 (0.5)	1.00–3.00
**Number of competitions**	1.73 (1.2)	1.00–4.00

SD = Standard deviation.

**Table 2:** Consumption of nutritional supplements (NS) by participants according to the Australian Institute of Sport’s (AIS) classification.

Group	Subcategories	Supplements	(n)	%
A	Sport Foods	Whey protein	46	96
		Bars	31	65
		EAA	31	65
		Non-whey protein	24	50
		Isotonic drink	27	56
	Medical supplements	Vitamin complexes	40	83
		Mineral complexes	28	58
		Vitamin D	26	54
	Performance supplements	Creatine	41	85
		Caffeine	36	75
		Beta-alanine	25	52
	Amino acids	BCAA	45	94
		EEAA	31	65
	Others	Omega-3	38	79
		L-Carnitine	34	71
		HMB	15	31
C		Arginine	35	73
		Magnesium	28	58
		CLA	28	58
		Testosterone booster	29	60
		Hormone precursor	25	52
		Chromium picolinate	21	44
		Ginseng	17	35
		Evening primrose oil	16	33
		Medium chain triglycerides	15	31
		Flax oil	14	29

BCAA: branched-chain amino acids; CLA: Conjugated linoleic acids; EAA: essential amino acids. * Data of Consumption of Group D are presented in Table 3.

**Table 3:** Consumption of banned substances.

Categories	Supplements	(n)	%
Stimulants	Ephedrine	32	66.7
	Pseudo-amphetamines	10	20.8
Prohormones and hormone Boosters	Anabolic steroids	35	72.9
	Insulin	30	62.5
	Testosterone enhancers	27	56
	Hormone precursors	24	50
	Growth hormones	11	22.9
Other	Diuretics	28	58

## References

[1] Sanchez-Oliver A.J., Martín-García C., Gálvez-Ruiz P., González-Jurado J.A. (2018). Mortality and economic expenses of cardiovascular diseases caused by physical inactivity in Spain. J. Phys. Educ. Sport.

[2] Myers J., Prakash M., Froelicher V., Do D., Partington S., Atwood J.E. (2002). Exercise capacity and mortality among men referred for exercise testing. N. Engl. J. Med..

[3] Jackson A.S., Sui X., Hébert J.R., Church T.S., Blair S.N. (2009). Role of lifestyle and aging on the longitudinal change in cardiorespiratory fitness. Arch. Intern. Med..

[4] Domínguez R., Maté-Muñoz J., Serra-Paya N., Garnacho-Castaño M. (2018). Lactate threshold as a measure of aerobic metabolism in resistance exercise. Int. J. Sports Med..

[5] Domínguez R., Garnacho-Castaño M.V., Maté-Muñoz J.L. (2016). Efectos del entrenamiento contra resistencias o resistance training en diversas patologías. Nutr. Hosp..

[6] Garber C.E., Blissmer B., Deschenes M.R., Franklin B.A., Lamonte M.J., Lee I.-M., Nieman D.C., Swain D.P., American College of Sports Medicine (2011). Quantity and quality of exercise for developing and maintaining cardiorespiratory, musculoskeletal, and neuromotor fitness in apparently healthy adults. Med. Sci. Sports Exerc..

[7] Lambert C.P., Frank L.L., Evans W.J. (2004). Macronutrient considerations for the sport of bodybuilding. Sports Med..

[8] Kim J.-H. (2018). The effects of daily food ingestion on improved immune functions and health promotion of bodybuilding athletes. J. Exerc. Rehabil..

[9] Gentil P. (2015). A nutrition and conditioning intervention for natural bodybuilding contest preparation: Observations and suggestions. J. Int. Soc. Sports Nutr..

[10] Fagerberg P. (2017). Negative consequences of low energy availability in natural male bodybuilding: A review. Int. J. Sport Nutr. Exerc. Metab..

[11] Mosley P.E. (2009). Bigorexia: Bodybuilding and muscle dysmorphia. Eur. Eat. Disord. Rev..

[12] Mäestu J., Eliakim A., Jürimäe J., Valter I., Jürimäe T. (2010). Anabolic and catabolic hormones and energy balance of the male bodybuilders during the preparation for the competition. J. Strength Cond. Res..

[13] Helms E.R., Aragon A.A., Fitschen P.J. (2014). Evidence-based recommendations for natural bodybuilding contest preparation: Nutrition and supplementation. J. Int. Soc. Sports Nutr..

[14] Martínez-Sanz J.M., Sospedra I., Ortiz C.M., Baladía E., Gil-Izquierdo A., Ortiz-Moncada R. (2017). Intended or unintended doping? A review of the presence of doping substances in dietary supplements used in sports. Nutrients.

[15] Baker J.S., Graham M., Davies B. (2006). Gym users and abuse of prescription drugs. J. R. Soc. Med..

[16] Perry P.J., Lund B.C., Deninger M.J., Kutscher E.C., Schneider J. (2005). Anabolic steroid use in weightlifters and bodybuilders: An internet survey of drug utilization. Clin. J. Sport Med..

[17] Athanasiadou I., Voss S., Lyris E., Aljaber A., Alsayrafi M., Georgakopoulos C. (2016). Analytical progresses of the World Anti-Doping Agency Olympic laboratories: A 2016 update from London to Rio. Bioanalysis.

[18] Puya-Braza J.M., Sánchez-Oliver A.J., da Silva S.F., Domínguez R. (2018). No hay diferencias en el consumo de suplementos nutricionales deportivos entre powerlifters internacionales y nacionales. J. Negat. No Posit. Results.

[19] Puya-Braza J.M., Sanchez-Oliver A.J. (2018). Consumo de suplementos deportivos en levantadores de peso de nivel nacional. Retos Nuevas Perspect. Educ. Física. Deport. y Recreación.

[20] Spendlove J., Mitchell L., Gifford J., Hackett D., Slater G., Cobley S., O’Connor H. (2015). Dietary intake of competitive bodybuilders. Sports Med..

[21] Westerman M.E., Charchenko C.M., Ziegelmann M.J., Bailey G.C., Nippoldt T.B., Trost L. (2016). Heavy testosterone use among bodybuilders: An uncommon cohort of illicit substance users. Mayo Clin. Proc..

[22] Ljungqvist A. (2017). Brief history of anti-doping. Med. Sport Sci..

[23] Sánchez Oliver A.J. (2013). Suplementación nutricional en la actividad físico-deportiva: análisis de la calidad del suplemento proteico consumido.

[24] Bird S.R., Goebel C., Burke L.M., Greaves R.F. (2016). Doping in sport and exercise: Anabolic, ergogenic, health and clinical issues. Ann. Clin. Biochem..

[25] De Hon O., Kuipers H., van Bottenburg M. (2014). Prevalence of doping use in elite sports: A review of numbers and methods. Sports Med..

[26] Stubbe J.H., Chorus A.M.J., Frank L.E., de Hon O., van der Heijden P.G.M. (2014). Prevalence of use of performance enhancing drugs by fitness centre members. Drug Test. Anal..

[27] LaBotz M., Griesemer B.A. (2016). Use of performance-enhancing substances. Pediatrics.

[28] Knapik J.J., Steelman R.A., Hoedebecke S.S., Austin K.G., Farina E.K., Lieberman H.R. (2016). Prevalence of dietary supplement use by athletes: Systematic review and meta-analysis. Sports Med..

[29] Australian Institute of Sport ABCD Classification System. https://www.sportaus.gov.au/__data/assets/pdf_file/0004/698557/AIS_Sports_Supplement_Framework_2019.pdf.

[30] Ostovar A., Haerinejad M.J., Farzaneh M.R., Keshavarz M. (2017). Adverse effects of performance-enhancing drugs on the kidney in the male bodybuilders. Sci. Sports.

[31] Haerinejad M.J., Ostovar A., Farzaneh M.R., Keshavarz M. (2016). The prevalence and characteristics of performance-enhancing drug use among bodybuilding athletes in the south of Iran, Bushehr. Asian J. Sports Med..

[32] Maughan R.J., Burke L.M., Dvorak J., Larson-Meyer D.E., Peeling P., Phillips S.M., Rawson E.S., Walsh N.P., Garthe I., Geyer H. (2018). IOC consensus statement: Dietary supplements and the high-performance athlete. Int. J. Sport Nutr. Exerc. Metab..

[33] Burd N.A., Tang J.E., Moore D.R., Phillips S.M. (2009). Exercise training and protein metabolism: Influences of contraction, protein intake, and sex-based differences. J. Appl. Physiol..

[34] Volpi E., Kobayashi H., Sheffield-Moore M., Mittendorfer B., Wolfe R.R. (2003). Essential amino acids are primarily responsible for the amino acid stimulation of muscle protein anabolism in healthy elderly adults. Am. J. Clin. Nutr..

[35] Wilkinson S.B., Tarnopolsky M.A., MacDonald M.J., MacDonald J.R., Armstrong D., Phillips S.M. (2007). Consumption of fluid skim milk promotes greater muscle protein accretion after resistance exercise than does consumption of an isonitrogenous and isoenergetic soy-protein beverage. Am. J. Clin. Nutr..

[36] Bemben M.G., Lamont H.S. (2005). Creatine supplementation and exercise performance: Recent findings. Sports Med..

[37] Phillips S.M. (2015). Nutritional supplements in support of resistance exercise to counter age-related sarcopenia. Adv. Nutr..

[38] Candow D.G., Chilibeck P.D. (2008). Timing of creatine or protein supplementation and resistance training in the elderly. Appl. Physiol. Nutr. Metab..

[39] Alves C.R.R., Santiago B.M., Lima F.R., Otaduy M.C.G., Calich A.L., Tritto A.C.C., de Sá Pinto A.L., Roschel H., Leite C.C., Benatti F.B., Bonfá E., Gualano B. (2013). Creatine supplementation in fibromyalgia: A randomized, double-blind, placebo-controlled trial. Arthritis Care Res. (Hoboken).

[40] Castillo D., Domínguez R., Rodríguez-Fernández A., Raya-González J. (2019). Effects of caffeine supplementation on power performance in a flywheel device: A randomised, double-blind cross-over study. Nutrients.

[41] Grgic J., Trexler E.T., Lazinica B., Pedisic Z. (2018). Effects of caffeine intake on muscle strength and power: A systematic review and meta-analysis. J. Int. Soc. Sports Nutr..

[42] Stellingwerff T., Anwander H., Egger A., Buehler T., Kreis R., Decombaz J., Boesch C. (2012). Effect of two β-alanine dosing protocols on muscle carnosine synthesis and washout. Amino Acids.

[43] Harris R.C., Tallon M.J., Dunnett M., Boobis L., Coakley J., Kim H.J., Fallowfield J.L., Hill C.A., Sale C., Wise J.A. (2006). The absorption of orally supplied β-alanine and its effect on muscle carnosine synthesis in human vastus lateralis. Amino Acids.

[44] Swietach P., Youm J.-B., Saegusa N., Leem C.-H., Spitzer K.W., Vaughan-Jones R.D. (2013). Coupled Ca2+/H+ transport by cytoplasmic buffers regulates local Ca2+ and H+ ion signaling. Proc. Natl. Acad. Sci. USA.

[45] Hoffman J., Ratamess N., Kang J., Mangine G., Faigenbaum A., Stout J. (2006). Effect of creatine and beta-alanine supplementation on performance and endocrine responses in strength/power athletes. Int. J. Sport Nutr. Exerc. Metab..

[46] Maté-Muñoz J.L., Lougedo J.H., Garnacho-Castaño M.V., Veiga-Herreros P., Del M., Lozano-Estevan C., García-Fernández P., De Jesús F., Guodemar-Pérez J., San Juan A.F., Domínguez R. (2018). Effects of β-alanine supplementation during a 5-week strength training program: A randomized, controlled study. J. Int. Soc. Sports Nutr..

[47] Carr B.M., Webster M.J., Boyd J.C., Hudson G.M., Scheett T.P. (2013). Sodium bicarbonate supplementation improves hypertrophy-type resistance exercise performance. Eur. J. Appl. Physiol..

[48] Duncan M.J., Weldon A., Price M.J. (2014). The effect of sodium bicarbonate ingestion on back squat and bench press exercise to failure. J. Strength Cond. Res..

[49] Thompson C., Vanhatalo A., Jell H., Fulford J., Carter J., Nyman L., Bailey S.J., Jones A.M. (2016). Dietary nitrate supplementation improves sprint and high-intensity intermittent running performance. Nitric Oxide.

[50] Domínguez R., Cuenca E., Maté-Muñoz J., García-Fernández P., Serra-Paya N., Estevan M., Herreros P., Garnacho-Castaño M., Domínguez R., Cuenca E. (2017). Effects of beetroot juice supplementation on cardiorespiratory endurance in athletes. A systematic review. Nutrients.

[51] Coggan A.R., Broadstreet S.R., Mikhalkova D., Bole I., Leibowitz J.L., Kadkhodayan A., Park S., Thomas D.P., Thies D., Peterson L.R. (2018). Dietary nitrate-induced increases in human muscle power: High versus low responders. Physiol. Rep..

[52] Coggan A.R., Leibowitz J.L., Kadkhodayan A., Thomas D.P., Ramamurthy S., Spearie C.A., Waller S., Farmer M., Peterson L.R. (2015). Effect of acute dietary nitrate intake on maximal knee extensor speed and power in healthy men and women. Nitric Oxide Biol. Chem..

[53] Domínguez R., Maté-Muñoz J.L., Cuenca E., García-Fernández P., Mata-Ordoñez F., Lozano-Estevan M.C., Veiga-Herreros P., da Silva S.F., Garnacho-Castaño M.V. (2018). Effects of beetroot juice supplementation on intermittent high-intensity exercise efforts. J. Int. Soc. Sports Nutr..

[54] Maughan R.J., Shirreffs S.M., Vernec A. (2018). Making decisions about supplement use. Int. J. Sport Nutr. Exerc. Metab..

[55] Deldicque L., Francaux M. (2016). Potential harmful effects of dietary supplements in sports medicine. Curr. Opin. Clin. Nutr. Metab. Care.

[56] Aquino L.F.M.C.D., Ribeiro R.D.O.R., Simoes J.S., Mano S.B., Mársico E.T., Conte Junior C.A. (2017). Mercury content in whey protein and potential risk for human health. J. Food Compos. Anal..

[57] Geller A.I., Shehab N., Weidle N.J., Lovegrove M.C., Wolpert B.J., Timbo B.B., Mozersky R.P., Budnitz D.S. (2015). Emergency department visits for adverse events related to dietary supplements. N. Engl. J. Med..

[58] Thomas D.T., Erdman K.A., Burke L.M. (2016). Position of the Academy of Nutrition and Dietetics, Dietitians of Canada, and the American College of Sports Medicine: Nutrition and athletic performance. J. Acad. Nutr. Diet..

[59] Rodriguez N.R., Di Marco N.M., Langley S., American Dietetic Association, Dietitians of Canada, American College of Sports Medicine (2009). American College of Sports Medicine position stand. Nutrition and athletic performance. Med. Sci. Sports Exerc..

[60] Domínguez R., Mata-Ordoñez F., Sánchez-Oliver A.J. (2017). Nutrición Deportiva Aplicada: Guía para Optimizar el Rendimiento.

[61] Mata-Ordoñez F., Sánchez-Oliver A.J., Domínguez Herrera R., Villegas García J.A. (2018). Suplementación en el deporte: Directrices desde la responsabilidad profesional. Habilid. Mot. Rev. ciencias la Act. física y del Deport..

